# Expression Analysis of Cytokine and Chemokine Genes during the Natural Course of Murine Experimental Autoimmune Uveoretinitis

**DOI:** 10.5402/2012/471617

**Published:** 2012-09-20

**Authors:** Noriyasu Hashida, Nobuyuki Ohguro, Kohji Nishida

**Affiliations:** Department of Ophthalmology, Osaka University Graduate School of Medicine, E7, 2-2 Yamadaoka, Suita, Osaka 565-0871, Japan

## Abstract

C57BL/6 mice were immunized with human interphotoreceptor retinoid-binding protein peptides to induce experimental autoimmune uveoretinitis (EAU). From the day of immunization to 30 days later, RNA was isolated daily from the mouse eyes. Dynamic changes in gene expression during the pathogenesis of EAU were analyzed by TaqMan gene expression assay that contained most chemokines/cytokines and their receptors, and signal transducer and activator of transcription (*STAT*) family genes, using beta-actin as the endogenous control. Gene clusters based on their expression profiles were analyzed to determine the candidate genes for the pathogenesis of inflammation. Hierarchical cluster analysis showed gene expression during EAU development in seven clustering patterns. Hierarchical cluster analysis also identified four distinct phases in daily expression: entrance, acceleration, deceleration, and remission. Gene expression changes in the EAU active phase showed synergetic upregulation of *Th1*-type genes *(IFN-gamma* and *CXCL10/IP-10*) with elevated *Th2*-type genes (*CCL17/TARC* and *IL-5*). Sequential expression changes of *STAT1*, *STAT6*, and *STAT3* genes represented the dynamic changes of *Th1, Th2*, and *Th17*-type inflammatory genes, respectively. The expression pattern of *STAT1* was representative of many gene movements. Our results suggested that coordinated action of *Th1, Th2*, and *Th17* genes and *STAT* family genes are involved in EAU development and resolution.

## 1. Introduction

Experimental autoimmune uveoretinitis (EAU) is a T-cell (CD4+)-mediated disease induced by inoculating the interphotoreceptor retinoid binding protein (IRBP) to genetically susceptible murine strains [1–4]. EAU is characterized by granuloma formation in the retina, retinal infiltration of polynuclear lymphocytes and macrophages, vasculitis, and destruction of photoreceptor cells [5–7]. EAU serves as an animal model of human uveitis (e.g., ocular sarcoidosis, Behcet's disease, or Vogt-Koyanagi-Harada disease) [2], [8]. Inflammation in the natural course of EAU begins about 9 days after peptide inoculation, reaches a peak, and spontaneously subsides afterward. Many investigators have reported the factors or cell types involved in the pathogenesis of EAU [9–15]. However, there is little information about the key factors that suppress excessive inflammation or are involved in the remission of EAU.

Increasing evidence suggests that expression changes in inflammatory genes exacerbate and cause relapses of ocular inflammation. Cytokines and chemokines play key roles in the recruitment of T-lymphocytes to inflammatory sites and are thought to be critical in the pathogenesis of EAU. Although the detailed mechanism governing the recruitment of T lymphocytes to the inflammatory site is unknown, various cytokines and chemokines have been implicated in regulating T-lymphocyte infiltration [9, 10]. In response to inflammation, the CXC chemokines CXCL10/IP-10 and CXCL9/Mig specifically attract T-effector cells via CXCR3, the cognate receptor for CXCL10/IP-10, CXCXL9/Mig, and I-TAC [10–12]. Bonecchi et al. showed that polarized Th1-type lymphocytes preferentially express CXCR3 and CCR5 [13]. Th1-type cytokines (*IL*-2, *IFN*-gamma) and chemokines/chemokine receptors (CXCL10, CXCR3) are upregulated in eyes with EAU, suggesting their contribution to the recruitment of Th1-type T cells into the eye during EAU development [14]. Recent studies have reported that Th17 cells are engaged in EAU development. Th17 cells are a newly discovered subtype of regulatory T-helper cells distinct from Th1 and Th2 cells that produce IL-17 and play pivotal roles in ocular inflammation [15].

Signal transducer and activator of transcription (STAT) proteins have been reported to have dual functions of signal transduction and activation of transcription [16]; however, little is known about their regulatory mechanism in EAU [15, 17, 18]. The major functions of STAT genes are related to the development of the immune system and the mediation of signaling form cytokine receptors. STAT3, one of STAT family protein, is reported to mediate the expression of a variety of genes in response to cell stimuli and apoptosis. STAT3 is an essential molecule required for the differentiation of the Th17-helper T cell, which has been implicated in a variety of autoimmune disorders, including EAU. STAT3 phosphorylation by JAK2 occurs via the binding of interleukin 6 family cytokines to the gp130 receptor [19], thus binding of cytokines and chemokines to their receptors and subsequent activation of STAT family genes play an important role in immune regulation.

There is a need to understand the expression of key player genes that control inflammation in EAU. However, sequential and comprehensive data analysis of cytokine and chemokine involvement in the development of ocular inflammation in EAU has not been performed. We previously investigated comprehensive gene expression analysis of inflammatory genes after systemic corticosteroid treatment using a hierarchical clustering method [20]. We also reported the usefulness of a method that analyzed the similarities in gene expression patterns and the application potential for analysis of time-space gene expression patterns. However, comparing the traditional approach of focusing on a limited number of genes at a time, the low-density array (LDA) system can globally identify targets by simultaneously investigating the quantitative expression changes in hundreds of genes. This system consists of quantitative reverse transcriptase-polymerase chain reaction (RT-PCR) system and calculates the expression data more precisely than microarray expression analysis [21]. We applied this system to learn about regulation of key gene expression during the natural course of EAU and determine a therapeutic target to control ocular inflammation.

In the current study, we investigated gene expression profiles during the natural course of EAU from peptide immunization through peak inflammation to remission of inflammation. We used a quantitative RT-PCR system specializing in cytokines/chemokines and their receptors and STAT family genes to identify which genes are targets in controlling excessive inflammation by focusing on the key genes using hierarchical clustering analysis.

## 2. Materials and Methods

### 2.1. Mice

Two hundred C57BL/6 mice were purchased from Japan SLC, Inc. (Hamamatsu, Shizuoka, Japan), housed under pathogen-free conditions in our animal facility at Osaka University, and used at 8 to 10 weeks of age. The treatment of animals conformed to the Association for Research in Vision and Ophthalmology Statement of the Use on Animals in Ophthalmic and Vision Research. The protocol was approved by the Committee on the Ethics of Animal Experiments of Osaka University (Permit number: 19-141-1).

### 2.2. Peptides and Adjuvants

A human IRBP epitope corresponding to 1 to 20 residues of IRBP sequences (GPTHLFQPSLVLDMAKVLLD) was selected to induce EAU in C57BL/6 mice as previously described [7, 22]. The peptide (purity, >95%) was purchased from Gene Net Co., Ltd. (Fukuoka, Japan). Complete Freund's adjuvant (CFA) and *Mycobacterium tuberculosis* H37Ra were purchased from Sigma-Aldrich (St. Louis, MO, USA) and DIFCO (Detroit, MI, USA), respectively. *Bordetella pertussis* toxin was purchased from Wako Pure Chemical Industries (Tokyo, Japan). 

### 2.3. Induction and Clinical Scoring of EAU

Three mice at each time point were used for gene expression assay. Ninety C57BL/6 mice were inoculated subcutaneously with 100 *μ*g IRBP in 0.2 ml emulsion with CFA (1/1 vol/vol) that had been supplemented with *M. tuberculosis* to a final concentration of 6.0 mg/ml. *B. pertussis* toxin (1-*μ*g) in 0.1 mL was intraperitoneally administered as an additional adjuvant. Ninety day-matched control mice were immunized with the same volume of phosphate buffered saline, instead of peptide, in CFA. Another 20 immunized mice were examined to assess the clinical EAU score at each time point. The clinical EAU scores (grades 0–4) [22, 23] were recorded and averaged daily with slit-lamp biomicroscopy from the beginning to 30 days after peptide immunization. 

### 2.4. Tissue Sampling and RNA Isolation

Whole eyes were prepared for RNA isolation every day from days 0 to 30. The globes were removed and prepared immediately for analysis. The eyes were treated in an RNase-free environment and frozen until analysis. The pooled eye was homogenized in tubes using the automated homogenization system (BioRobot EZ1 Systems, Qiagen, Valencia, CA, USA). Total RNA from the eyes was extracted using a kit (EZ1 RNA Tissue Mini Kit, Qiagen) with an additional DNase digestion step, according to the manufacturer's protocol. A quality check of isolated RNA was performed as described previously [20, 24]. The RNA samples obtained from two eyes of one mouse were processed together. Six eyes of three mice at each time point were used for the gene expression assay.

### 2.5. Description of LDA

LDA (Applied Biosystems Inc. [ABI], Foster City, CA, USA) is a card with the selected TaqMan gene expression assays preloaded into each reaction well (TaqMan LDAs, ABI). The card contains a preset assays-on-demand assay mix of primers and FAM dye-labeled TaqMan minor groove binding probes for 96 user-selected target genes used for real-time PCR measurements. LDA is a research tool for profiling gene expression using the comparative Ct method of relative quantification [25, 26]. The PRISM 7900HT Sequence Detection System (ABI) also supports real-time relative quantification of nucleic acids using the ddCt method. A description of the genes selected for quantitative PCR analysis is available in Supplementary Table  1 of the Supplementary Material available online at doi: 10.5402/2012/471617. In gene selection, we referred to the microarray data described previously. The selected genes were the top 100 genes that were significantly up-regulated more than twofold during microarray analysis [20]. We also included *STAT1*, *STAT3*, *STAT4*, and *STAT6*, members of the STAT family of transcription factors for analyzing the Th1-, Th2-or Th17-type transcription factors [19, 27]. Finally, because of the limitations of commercially available primers and probe sets, we selected 96 genes including beta-actin as negative controls for expression analysis. Three independent experiments were performed for data verification. The results of triplicate analysis of each sample were consistent.

### 2.6. Quantitative RT-PCR

An RNA sample (800 ng) derived from the indicated time points was reverse transcribed with the commercially available kit (High Capacity Kit, ABI) to synthesize double-stranded cDNA, according to the manufacturer's instructions. Gene-specific PCR products were measured continuously using the PRISM 7900HT Sequence Detection System according to the manufacturer's protocols. Relative levels of gene expression were determined from the fluorescence data generated during PCR using the PRISM 7900HT Sequence Detection System, a relative quantification system, and its software. All reactions were performed with primer-probe assays as follows: incubation for 2 minutes at 50°C and 10 minutes at 94.5°C, the 40 PCR cycles at 97°C for 15 seconds, and annealing/extension at 59.7°C for 1 minute. Three independent experiments were performed to compare the values of the control and subject groups. Relative quantification was performed with the SDS2.1 software (ABI) in the PRISM 7900HT Sequence Detection System. Normalization was obtained using beta-actin as an endogenous control and day-matched control murine eyeballs as calibration samples compared to the peptide-injected group. The results are expressed as fold increases.

### 2.7. Hierarchical Clustering Method

To study the relationship between individual data sets, we used hierarchical clustering methods [28] visualized using the DNASIS Stat (Hitachi Software Engineering Co., Ltd., Tokyo, Japan) according to the manufacturer's instructions as described previously [29]. The Silhouette index and Dunn's index in the clustering analysis were used to determine the clustering numbers. The relationship is presented graphically in a dendogram, in which the closer individual groups are, the more the expression profiles resemble one another.

## 3. Results 

### 3.1. Clinical Assessment and Scoring of EAU Mice

**Figure 1:**
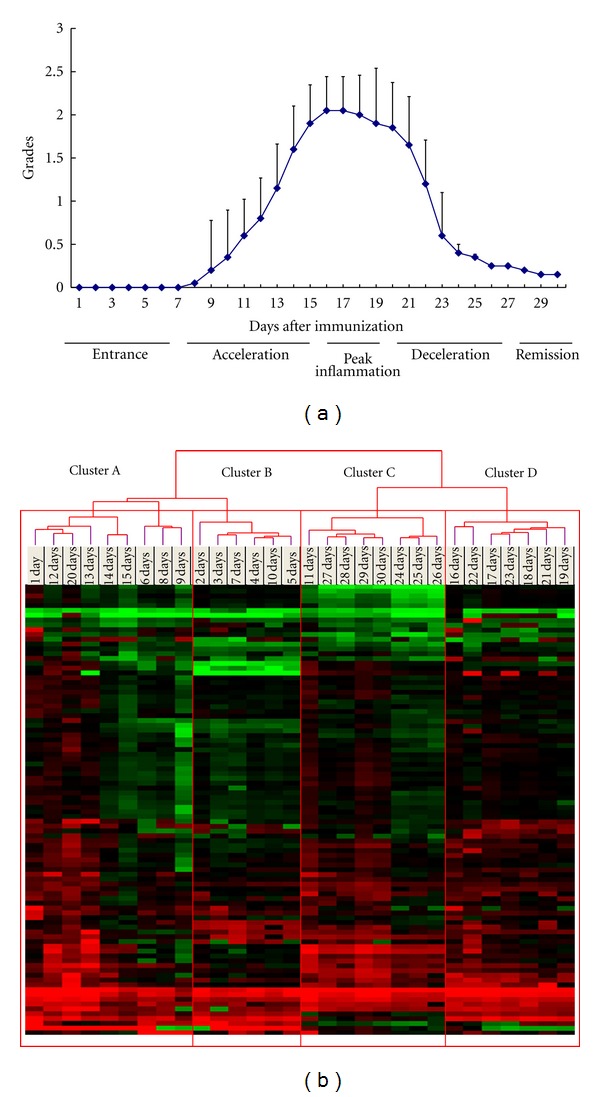
Clinical assessment and cluster analysis of daily expression of inflammatory genes in EAU. (a) Clinical scores of 20 C57BL/6 mice with EAU. The clinical scores begin to increase from 8 to 16 days of peak inflammation and subside from 20 to 30 days. The clinical scores are calculated from the average scores of the 20 EAU mice ± SD. Days 1 to 7 with no inflammation represent the entrance phase, days 8 to 15 with the increase to maximum inflammation represent the acceleration phase, days 16 to 19 with active peak inflammation represent the peak inflammation phase, days 20 to 26 with decreased severity of EAU represent the deceleration phase, and days 27 to 30 with subsiding ocular inflammation represent the remission phase. (b) The dendogram shows hierarchical cluster analysis of daily expression profiles. The individual samples are clustered in branches of the dendogram based on overall similarity in the pattern of gene expression. The groups of genes in each cluster are as follows: entrance, cluster B; acceleration, cluster A; deceleration, cluster D; remission, cluster C.

Figure 1(a) shows the sequential changes in the clinical EAU scores from the beginning of immunization to 30 days in 20 mice with EAU. The EAU scores ranged from grades 1 to 3 [22, 23]. The peak inflammatory response was observed 16 ± 1 days after peptide immunization, consistent with a previous study [30]. The clinical EAU scores began to decrease 20 days after immunization, drastically declined thereafter, and subsided 27 days after inoculation (Figure 1(a)). Based on the clinical EAU course, we divided the entire EAU period into five phases: entrance (days 1 to 7), acceleration (days 8 to 15), peak inflammation (days 16 to 19), deceleration (days 20 to 26), and remission (days 27 to 30).

### 3.2. Clustering of Gene Expression Patterns Based at Various Time Points after Immunization during EAU (Daily Expression Basis Analysis)

A complete list of probe sets and expression data for each day are shown in Supplementary Table  2. Clustering analysis clearly discriminated four distinct clusters in daily expression (Figure 1(b)). Cluster A contained days 1, 6, 8, 9, 12 to 15, and 20. Days 6, 8, 9, and 12 to 15 formed a cluster that corresponded approximately to acceleration, except for days 1 and 20. Cluster B included days 2, 3 to 5, 7, and 10 that corresponded approximately to the entrance phase. Cluster C included days 11 and 24 to 30; days 24 to 26 formed a small more detailed cluster that corresponded to remission and the end of deceleration, except for day 11. Cluster D included days 16 to 19 and 21 to 23 and corresponded completely to peak inflammation and the start of deceleration, except for day 20. Although some parts did not exactly match the clinical phase, we concluded that analysis of the daily expressions correlated well with the clinical scoring analysis.

### 3.3. Clustering of Gene Expression Patterns Based on Genes during the EAU Course (Gene-Based Analysis)

**Figure 2:**
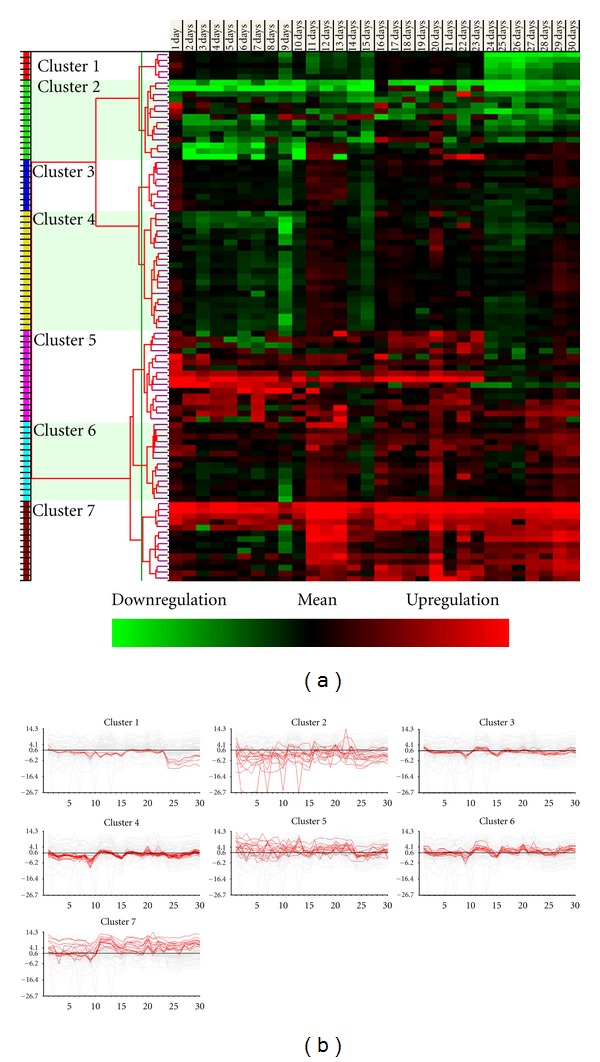
Gene-based clustering analysis of inflammatory genes during the EAU course. (a) The cluster images from an average of three independent experiments. Each row represents individual genes and each column an individual sample. The data are depicted according to the color scale. The horizontal hierarchical trees show the degree of similarity in gene expression patterns among each data set. The genes are clustered into seven groups according to the highest score on the Silhouette index. (B) The graphs show the representative gene expression profiles in corresponding clusters 1 to 7. The graphs show the 2^*x*^-fold changes of each inflammatory gene and the days after immunization.

We then focused on the sequential expression changes of each inflammatory gene and performed hierarchical clustering analysis. The optimum cluster number was determined by calculating the scores of the Silhouette index. The genes ultimately were clustered into seven groups according to the highest index score.  Figure 2(a) shows all images of hierarchical clustering analysis. The cluster images of different classes of gene expression profiles are displayed as horizontal strips. 

Cluster 1 contained five genes (*CCL11*, *IL-10rb*, *IL-18*, *IL-13ra1*, and *IL-1rap)*. The gene expression level in this cluster was almost constant until day 24 and downregulated thereafter (Figure 2(b)). Cluster 2 contained 14 genes (representative genes, *IL-4*, *IFN-beta*, and *STAT6)*, most of which were down-regulated with substantial fluctuations during observation. Clusters 3 and 4 contained nine and 21 genes, respectively; the gene expression levels in these two clusters were almost constant throughout observation, with down-regulation on days 9 and 15. In cluster 4, upregulated gene expression also was observed on day 20. The representative genes in cluster 3 were *CCL4/MIP-1beta* and *IL-11*, and in cluster 4 *TNF-alpha*, *IL-15*, and *CX3CR1/Fractalkine*. Cluster 5 contained 16 genes that were upregulated with fluctuating changes; this cluster contained *CCL17*,* STAT3, IL-6*, and *IL-17*. Cluster 6 contained 13 genes with constant expression up to day 9, transient down-regulation on day 9, upregulation over the following several days, slight down-regulation on day 15, transient upregulation on day 20, and almost constant expression thereafter. This cluster contained *IL-10* and *IL-5*, which are involved in the Th2-type immune response. Cluster 7 contained 15 genes, the expression pattern of which resembled cluster 6, although it was more dynamic. The representative genes in this cluster were *STAT1*, *STAT4*, *CXCL9/Mig*, and *IFN-gamma* that are involved in Th1-type immunologic responses in EAU. Supplementary Table  3 shows the detailed information for each cluster.

### 3.4. Sequential Expression Changes of Inflammatory Genes in the Natural EAU Course

**Figure 3:**
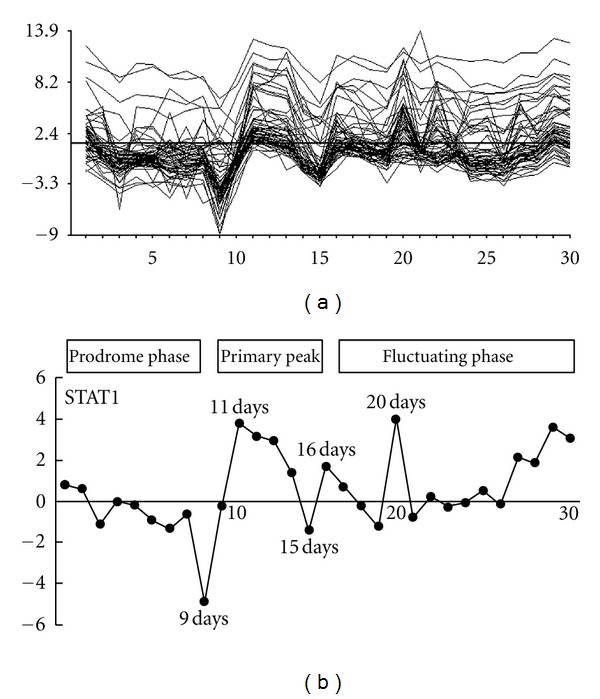
*STAT1* gene had a representative motif of all data from the four selected clusters. (a) Sequential expression changes in inflammatory genes selected from clusters 3, 4, 6, and 7. The graph shows the 2^*x*^-fold changes of each inflammatory gene and the days after immunization. Dynamic expression changes are seen from peptide immunization to day 30. The graph shows several turning points before the EAU peak and fluctuating changes after the EAU peak. (b) The *STAT1 *gene expression pattern is representative of all data from the four selected clusters. The expression patterns are divided into the prodrome, primary peak, and fluctuating phases.

Since daily analysis showed that gene expression patterns were well correlated with the EAU clinical course, we selected clusters 3, 4, 6, and 7, which had unique sequential gene expression changes from gene basis analysis. We excluded cluster 1 because the unique expression changes occurred after inflammation subsided. The graph indicated that gene expression changed dramatically from the beginning of immunization.  Figure 3(a) plots all data from these four clusters and indicates three turning points, days 9, 15, and 20. Day 9 was early in the accelerated phase, day 15 immediately preceded the peak inflammatory phase, and day 20 was the start of deceleration.  Figure 3(b) shows the *STAT1* gene expression pattern. Figures 3(a) and 3(b) show that the expression pattern of the *STAT1* gene had a representative motif of all data from the four selected clusters.

### 3.5. Gene Comparison of Upregulated Genes on Days 1 and 20

Figure 1(b) shows that almost all genes studied were significantly up-regulated on day 1, which was distinctly different from days 2, 3, 4, and 5. This suggested that gene expression changes on day 1 resulted from systemic stress caused by immunization, and not the EAU clinical course. However, our data clearly indicated that day 20 was one of the most important turning points in the clinical course and gene expression changes during EAU. Since widely separated days in the EAU course, days 1 and day 20, were in the same cluster, we did further gene expression analysis on those days.  Table 1 shows the list of the top 20 up-regulated genes on days 1 and 20, and nine genes were common to both days. Th1-type inflammatory genes such as *INF-gamma* and *CXCR3*, which are up-regulated in EAU, and *CCL17/TARC*, which is involved in Th2-type immune responses were included in each group. At different points, the proinflammatory cytokine *IL-2* was up-regulated only on day 1, and *CXCL10/IP-10* and *CXCR6/BONZO* corresponding to T-cell-specific responses were up-regulated only on day 20. The down-regulated genes in both groups were unlikely to have any relation to the Th1/Th2-type inflammatory genes (data not shown).

### 3.6. Daily Expression Changes in STAT Family Genes

Since previous studies [31–33] reported that a balance between Th1 and Th2 is important in EAU development, we focused on the daily expression changes in STAT family genes, *STAT1, STAT4,* and *STAT6*. We also checked the expression of *STAT3*, which is an important molecule for Th17-type immune responses.

**Figure 4:**
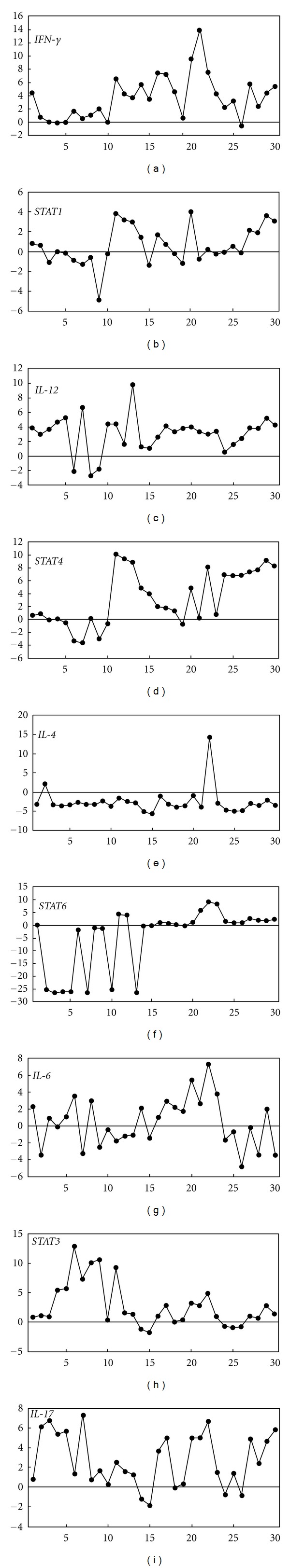
Daily expression changes in STAT family genes in EAU. The sequential expression changes of (a) *IFN-*gamma, (c) *IL-12*, (e) *IL-4*, and their corresponding signaling molecules (b) *STAT1*, (d) *STAT4*, and (f) *STAT6* are superimposed, respectively. The graphs show the 2^*x*^-fold changes of each inflammatory gene and the days after immunization. Expression patterns are strongly correlated with each other.

*STAT4 *is critical for the response to *IL-12*, *STAT1* in the response to *IFN-gamma*, *STAT3 *is crucial for *IL-6 *signaling, and *STAT6* is a central mediator of *IL-4*. For example, representative Th1 molecules *STAT1* and *STAT4*, belonged to same clusters, their gene expression patterns were similar. The level of gene expression of these molecules was low until the middle of the acceleration phase. However, the expression was suddenly up-regulated on day 11, gradually declined thereafter, and suddenly up-regulated on day 20, the beginning of deceleration (Figures 4(b) and 4(d)). The responsible cytokines, *IL-12* and *IFN-gamma*, showed slightly different expression patterns (Figures 4(a) and 4(c)). *IL-12 *showed a constantly high expression except for a few days early in the acceleration phase. *IFN-gamma* was up-regulated from days 11 to 18 (from mid-acceleration to peak inflammation). Extremely high expression of *IFN-gamma *was seen around day 20 (beginning of deceleration). In contrast, *STAT6* and *IL-4*, Th2-type immune responses, belonged to cluster 2. *IL-4* showed unremarkable expression changes through the observation period except for day 22, when it was significantly up-regulated. *STAT6* had down-regulated or unchanged expression until day 22 but showed significantly high expression thereafter (Figures 4(e) and 4(f)). Nest,* STAT3*, *IL-6,* and* IL-17*, Th17-type genes, belonged to cluster 5. The genes in cluster 5 displayed fluctuating expression pattern from the beginning of peptide inoculation. For example, *IL-17* was up-regulated during entrance phase and *STAT3* was up-regulated during acceleration phase(Figures 4(g), 4(h), and 4(i)). 

## 4. Discussion

Many investigators have attempted to identify the key factors in EAU development and remission; however, most focused on selected genes and selected days in the course of EAU. We collected sequential data from the beginning of immunization to the end of inflammation and comprehensive gene expression data in the natural course of EAU using the LDA system. We also performed hierarchical clustering analysis of the data based on daily expression and gene-based analysis and found four clusters of days in daily analysis (Figure 1) and seven clusters of genes in gene-based analysis (Figure 2). The current study is the first report to present an overall picture of gene expression changes in EAU.

It is reasonable that the results of daily hierarchical clustering analysis correspond to the entire clinical phase, although the argument remains about whether this is a cause or result. However, a few days did not match the clinical phase, notably, days 1 and 20; days 1 and 20 were in cluster A, which included acceleration (days 12–15). Expression changes on day 1 could be affected directly by CFA and *B. pertussis* toxin immunization. Accelerated immunoreactions that occurred on day 1 reflect the immunologic status of the entire body. Therefore, the genes on day 1 formed the same cluster with cluster A. Under our experimental conditions, EAU inflammation began to subside on day 20. It was noteworthy that the genes on days 20 (start of deceleration) displayed expression patterns similar to those during acceleration. Considering that eyes also are affected by immunologic stress in the entire body, gene up-regulation on day 20 should reflect body-wide stress. The reaction to resolution of substantial stress resembles the movements to weaken inflammation. Therefore, transient overexpression of many genes on day 20 should play an important role in EAU remission. Few previous reports have mentioned EAU remission. In the current results, gene expression changes around day 20 should drive the active EAU stage to remission. We agreed with the observation that EAU inflammation spontaneously subsided after peak inflammation [22, 23, 30]. Considering the sequential events during EAU remission, there should be a turning point from active ocular inflammation to remission. We speculated about two possibilities. One is that the genes in cluster A were responsible for the stress. The second was that the same group of genes might be engaged in inflammatory development and remission. Further study is necessary to prove this.

Previous studies reported the predominance of Th1-type lymphocytes and the association of chemokines and chemokine receptors in EAU pathogenesis [14, 31]. We showed the high expression of Th1-type genes such as *CXCL9/Mig* and *CXCR3* during active EAU (Supplementary Table  3). These genes were classified into cluster 7. Moreover, the pathogenesis of EAU has been related to elevated Th1-type cytokines during the active phase [4, 32, 33]. We confirmed these expressions in the genes of Th1-type cytokine IL-12 and IFN-gamma (Figures 4(a) and 4(c)). Several researchers have reported an association of Th2-type cytokines with EAU resolution [33, 34]. For example, *IL*-10 mRNA expression in an eye with EAU coincided with EAU resolution [35, 36]. We also found transient up-regulation of *IL-5*, *IL-10*, and *CCL17/TARC* during peak inflammation and remission (Supplementary Table  3). *IL-5* and *IL-10* genes interestingly were in cluster 6. Considering Th2-type immunoresponses may occur even during acute EAU, we conclude that Th2-type immunoresponses and IL-10 expression play an important role in EAU remission. Su et al. reported that Th2- and Th17-type immune responses are relatively activated in IFN-gamma knockout mice with a depletion of Th1-type T-cells, suggesting IFN-gamma involvement in the pathogenesis and remission of EAU [37]. Based on this report and our observation, simultaneously upregulated Th1 responses, as seen in day 20 with extremely high *IFN-gamma*, might lead to suppress Th17-type immune responses. Considering *IFN-gamma* involvement in ocular inflammation, as shown in Figure 3, it is reasonable that expression pattern of the *STAT1* gene had a representative motif of most of the genes.

Considering the expression changes of all genes (Figure 3), the expression pattern of the STAT family gene seems to represent many genes with various movements. *STAT1 *is activated by a number of different ligands, including *IFN-alpha*, *IFN-gamma*, and epithelial growth factor. IFN-gamma stimulation induces phosphorylation of *STAT1* and promotes formation of STAT1 homodimers, resulting in many gene transcriptions. *STAT1* plays a necessary role and dedicated role in mediating IFN-dependent biologic responses in EAU. Because effector T cells in EAU possess a Th1-type phenotype, it seems reasonable that Th1-prone cytokines such as *INF-gamma* and *IL-12* induced the inflammation of EAU and expression patterns of corresponding signaling molecules *STAT1* and *STAT4 *represented all gene movements. The expression patterns of *IL-4* and *STAT6*, which belonged to the same cluster, were remarkable. These genes could not be detected in the eye before and during acute EAU, and transient overexpression was observed on day 22. High expression in the late phase was consistent with a previous study, although in a different model [33]. We speculated that activation of Th2-type T cells that express *STAT6* and *IL-4* might be involved in maintaining inflammation during the EAU natural course. The expression pattern of *IL-6*, *IL-17*, and *STAT3*, that played important role in Th17-type T-cell differentiations and activations [38], belonged to the same cluster. Especially, up-regulation of *IL-17* at the early stage of entrance phase should imply that activation of Th17-type T-cell is necessary for the onset of EAU. Taking all things together, hierarchical cluster analysis clearly discriminated the expression profiles of each STAT family gene and relevant cytokine genes, suggesting that clinical stages of EAU are determined and driven by the expression changes of STAT family genes.

EAU displayed a monophasic disease course that peaked about 2 weeks after immunization followed by resolution when assessed histologically. However, our results showed that many gene expression patterns fluctuated up and down. Kerr et al. found that cells that infiltrated the retina in EAU peaked about 2 weeks after immunization and retinal leukocytes fluctuated after peak inflammation [39]. These authors also reported three distinct phases: the prodrome phase that occurred after immunization with CFA and pertussis toxin, the primary peak that corresponded to active EAU inflammation, and the secondary regulatory phase in which multiple peaks in numbers of infiltrating cells were observed. Considering the sequential events in ocular inflammation, the fluctuating gene expression patterns in the current results could reflect fluctuating retinal infiltrates in eyes with EAU. Analysis of fluctuating expression changes in genes would lead to an understanding of human uveitis, which also follows relapsing and remitting clinical courses.

In the current study, we showed simultaneous up-regulation of *Th1*-and *Th2*-type genes and *STAT* gene involvement in EAU development and resolution. We also identified sequential expression changes of STAT family genes not previously reported. In any event, the pronounced diversity of inflammatory genes during the EAU natural course defines an important mechanism of ocular inflammation. It remains unclear which cluster manages ocular inflammation; however, the marked diversity of inflammatory genes implies that they have key roles in controlling the characteristics of ocular inflammation and EAU resolution.

## Supplementary Material

Supplementary table 1: Description of selected cytokine and chemokine genes.
Supplementary table 2: x-fold changes of each inflammatory gene and the day after immunization.
Supplementary table 3: Clustering analysis data of 2^x^-fold changes of each inflammatory genes.

## Figures and Tables

**Table 1:** Top 20 upregulated genes.

1 day		20 days	
*Ccr3*	2599.9	*Il9*	4015.4
*Il9*	1334.1	*Ccr4*	2818.3
*Il2*	1210.1	*Cxcl9*	1692.6
*Il8rb*	852.2	*Cxcr3*	1444.5
*Csf2rb1*	418.3	*Il8rb*	1040.4
*Ccr8*	370.6	*Ifng*	768.5
*Cxcl2*	315.2	*Xcl1*	662.8
*Xcr1*	304.8	*Csf2rb1*	508.7
*Il2rb*	244.2	*Il12rb2*	402.3
*Ccr7*	195.6	*Ccl17*	262.1
*Ifnb*	47.2	*Cxcl2*	231.6
*Il5*	34.2	*Ccr7*	163.1
*Ccl17*	31.9	*Cxcl10*	92.4
*Ifng*	22.1	*Cxcr6*	65.6
*Ccl20*	21.3	*Il2rb*	60.6
*Cxcr3*	20.7	*Ccl20*	56.6
*Csf3*	20.6	*Il7r*	56.5
*Il10*	20.0	*Ccl7*	55.0
*Il12rb2*	16.4	*Csf2rb2*	51.0
*Il18*	14.9	*Il6*	45.7
